# Protein Kinase C-α Is a Gatekeeper of *Cryptosporidium* Sporozoite Adherence and Invasion

**DOI:** 10.1128/iai.00679-21

**Published:** 2022-03-17

**Authors:** Sayo McCowin, William A. Petri, Chelsea Marie

**Affiliations:** a Department of Medicine, Infectious Diseases and International Health, University of Virginia School of Medicine, Charlottesville, Virginia, USA; b Department of Pathology, University of Virginia School of Medicine, Charlottesville, Virginia, USA; c Department of Microbiology, Immunology and Cancer Biology, University of Virginia School of Medicine, Charlottesville, Virginia, USA; University of California, Davis

**Keywords:** *Cryptosporidium*, adherence, invasion, actin cytoskeleton, PKCα, susceptibility, *PRKCA*

## Abstract

*Cryptosporidium* infection is a leading cause of diarrhea-associated morbidity and mortality in young children globally. Single nucleotide polymorphisms (SNPs) in the human protein kinase C-α (*PRKCA*) gene region have been associated with susceptibility to cryptosporidiosis. Here, we examined the role of protein kinase C-α (PKCα) activity in human HCT-8 intestinal epithelial cells during infection with Cryptosporidium parvum sporozoites. To delineate the role of PKCα in infection, we developed a fluorescence-based imaging assay to differentiate adherent from intracellular parasites. We tested pharmacological agonists and antagonists of PKCα and measured the effect on C. parvum sporozoite adherence to and invasion of HCT-8 cells. We demonstrate that both PKCα agonists and antagonists significantly alter parasite adherence and invasion *in vitro*. We found that HCT-8 cell PKCα is activated by C. parvum infection. Our findings suggest intestinal epithelial cell PKCα as a potential host-directed therapeutic target for cryptosporidiosis and implicate PKCα activity as a mediator of parasite adherence and invasion.

## INTRODUCTION

*Cryptosporidium* spp. are a family of obligate intracellular parasites with increasing importance in global health. Although there are over 20 species relevant to humans, Cryptosporidium parvum, C. hominis, and C. meleagridis are responsible for the vast majority of infections (1–3). *Cryptosporidium* infection causes diarrhea that can be life-threatening, and in immunocompromised individuals (e.g., HIV/AIDS), this diarrhea can be prolonged. The burden of infection is most severe in low- and middle-income countries, with cryptosporidiosis being classified as the second leading cause of diarrhea-associated mortality in young children (4, 5). In addition to morbidity and mortality, pediatric cryptosporidiosis is associated with malnutrition and long-term developmental deficits (6–8). At present, no vaccine is available for cryptosporidiosis, and the only approved drug, nitazoxanide, has poor efficacy in immunocompromised patients and malnourished children (9, 10).

During human infection, *Cryptosporidium* oocysts excyst in the upper small intestine, presenting four sporozoites with gliding motility that interact with the mucosal epithelium (11, 12). These motile sporozoites attach to the host epithelium through interactions with host cell surface ligands (13) and sporozoite surface lectins (14, 15). Upon interaction, glycoproteins on the surface of the host cell membrane initiate a signaling cascade aggregating filamentous actin (F-actin) to the site of sporozoite contact and form a host membrane protrusion resulting in the encapsulation of the sporozoite (16–18). Henceforth, *Cryptosporidium* exists in an intracellular yet extracytoplasmic vacuole formed by and partitioned off from the host cell by F-actin (19). The formation of this F-actin pedestal at the site of infection has been directly linked to the actin-associated signaling pathways of the neural Wiskott-Aldrich syndrome protein (N-WASP) and the Arp2/3 complex (20). The membrane protrusions encapsulating *Cryptosporidium* during invasion are additionally dependent on localized volume increases mediated by glucose and water influxes (21, 22). The recruitment of Na^+^/glucose cotransporter (SGLT1) and aquaporin 1 (AQP1) to the site of infection contributes to this actin-dependent cellular invasion. Analysis of the C. parvum transcriptome during development has identified the expression of stage-specific genes over the course of infection (23–25). Paired with microscopy imaging, a timeline for *Cryptosporidium* development and propagation *in vitro* has been well characterized. This entire life cycle has been modeled *in vitro* primarily using immortalized cell lines (26). However, the complete host cell repertoire that is usurped by *Cryptosporidium* spp. to achieve this has not yet been described.

Many studies have identified factors involved in *Cryptosporidium* infection on both sides of the parasite-host interface (18, 27, 28). Recently, we found that variation within the *PRKCA* gene was associated with increased susceptibility to cryptosporidiosis in children (29). Here, we asked the question, Is human protein kinase C-α (PKCα) mechanistically involved in *Cryptosporidium* infection *in vitro*? In a recent study, a high-throughput phenotypic screen identified a selective protein kinase C-α/β1 (PKCα/β1) inhibitor, Gö6976, as having potent anticryptosporidial activity *in vitro* (30). Gö6976 reduced infection of HCT-8 cells with both C. parvum and C. hominis at nanomolar doses with an EC_50_ (half-maximal effective concentration) for inhibition of C. parvum infection coinciding with the reported Gö6976 half-maximal inhibitory concentration (IC_50_) for PKCα (31). Furthermore, HCT-8 cell *PRKCA* mRNA expression has been shown to be significantly decreased after C. parvum infection compared to controls (32). From this evidence, we focused on exploring human intestinal epithelial cell PKCα further during *Cryptosporidium* infection.

In this study, we investigated the role of human intestinal epithelial cell PKCα during C. parvum infection *in vitro*. PKCα has been implicated in the regulation of host actin cytoskeletal remodeling, a requirement of parasite invasion; therefore, we targeted PKCα activity during this event. We found that PKCα mediated the earliest stages of C. parvum infection, sporozoite adherence and invasion, in human intestinal epithelial cells. Additionally, host PKCα was activated during sporozoite invasion. Our work exposes PKCα as a potential anticryptosporidial host target through inhibition of C. parvum adherence and invasion. Collectively, this work advances the understanding of *Cryptosporidium* pathogenesis and links a host protein to early infection.

## RESULTS

### C. parvum activates host PKCα during invasion.

**FIG 1:**
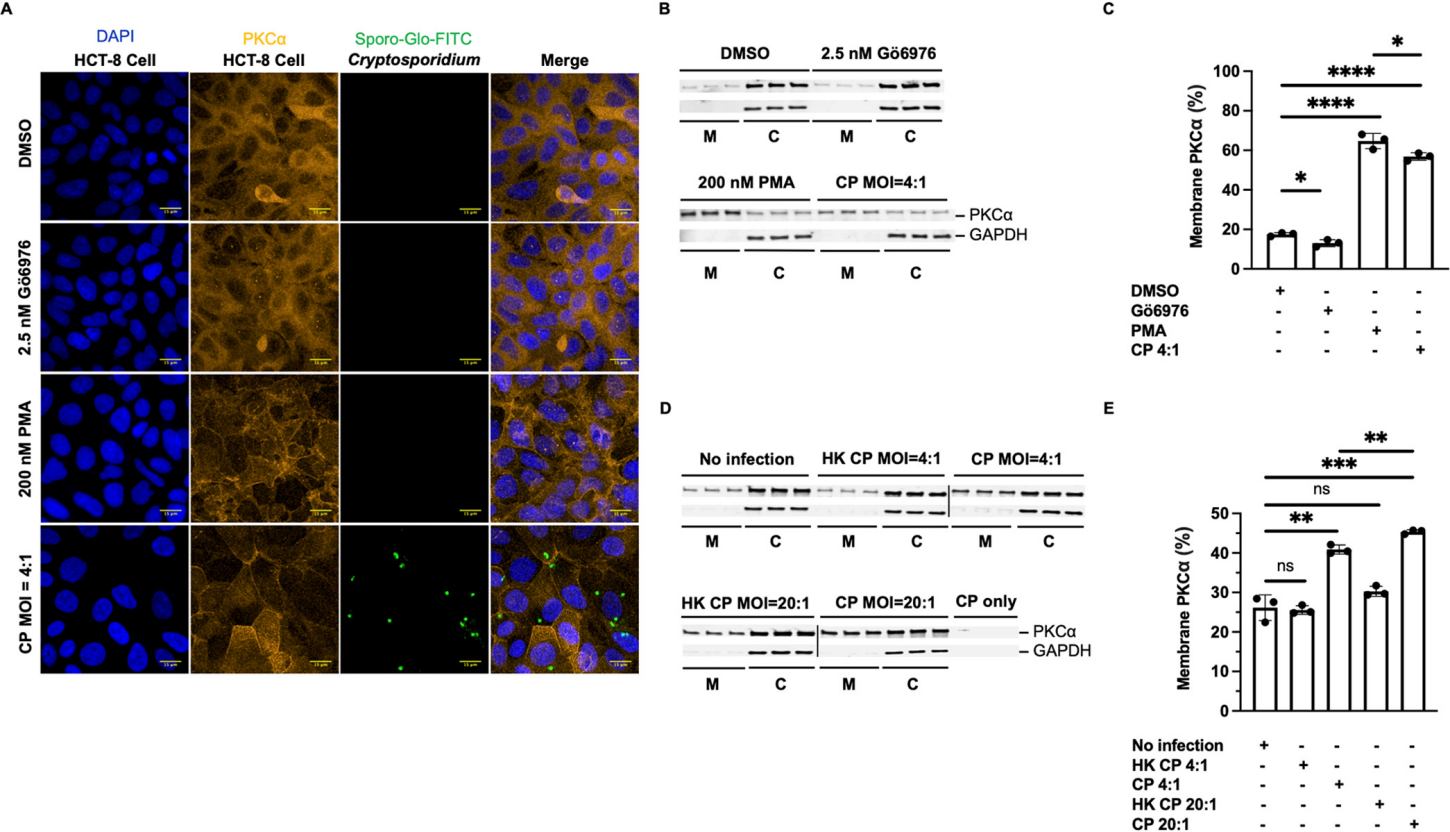
C. parvum activates host PKCα during invasion. (A) Intestinal epithelial cells were treated with DMSO, 2.5 nM Gö6976, 200 nM PMA, or C. parvum (CP) at an MOI of 4:1 as described in Materials and Methods. High-magnification (63× objective) confocal fluorescence microscopy images of DAPI (HCT-8 cell nucleus), anti-PKCα-Cy3 (PKCα), FITC-conjugated Sporo-Glo (total C. parvum), and merged channels are shown. Data from one of two independent experiments are shown. (B) Intestinal epithelial cells were treated with DMSO, 2.5 nM Gö6976, 200 nM PMA, or C. parvum at an MOI of 4:1. Immunoblots of cell membrane (M) and cytoplasmic (C) fractions are displayed in biological triplicate (*n* = 2). (C) Bar graph depicting quantification of membrane-associated PKCα (activation) after exposure to treatment. (D) Intestinal epithelial cells were treated with infection medium, heat-killed (HK) C. parvum at an MOI of 4:1, live C. parvum at an MOI of 4:1, HK C. parvum at an MOI of 20:1, or live C. parvum at an MOI of 20:1 for 2 h. Immunoblots of cell membrane and cytoplasmic fractions without C. parvum or with HK C. parvum at an MOI of 4:1, live C. parvum at an MOI of 4:1, HK C. parvum at an MOI of 20:1, and live C. parvum at an MOI of 20:1 are displayed in biological triplicate (*n* = 2). (E) Bar graph depicting quantification of membrane-associated PKCα (activation) after exposure to treatment. Asterisks denote the results of one-tailed unpaired Student’s *t* test used to analyze PKCα membrane association (*, *P* < 0.05; **, *P* < 0.01; ***, *P* < 0.001; ****, *P* < 0.00001; ns, not significant).

We investigated PKCα localization as a measure of activation during C. parvum invasion of HCT-8 cells. After binding the requisite cofactors for activation (phosphatidylserine, calcium, and diacylglycerol), activated PKCα translocates from the host cytoplasm to the membrane (33). We used a traditional *in vitro* fluorescence-based microscopy assay to simultaneously visualize C. parvum and intestinal epithelial cell PKCα. As a positive control for the activation of PKCα, we treated HCT-8 cells with phorbol-12-myristate 13-acetate (PMA). PMA is a well-established activator and induces the translocation of PKC to the cell membrane, where it is tightly bound and highly active (34–36). As predicted, exposure to 200 nM PMA for 1 h caused ubiquitous PKCα colocalization with the host membrane (Fig. 1A). Contrarily, we observed a decrease in membrane-associated PKCα when HCT-8 cells were treated with the PKCα inhibitor Gö6976 (2.5 nM) for 1 h (Fig. 1A). When HCT-8 cells were infected with C. parvum sporozoites at a multiplicity of infection (MOI) of 4:1 (sporozoite/cell ratio) for 2 h, we observed an increase in membrane-associated PKCα (Fig. 1A). Notably, the increase in C. parvum-induced membrane-associated PKCα is not limited to the *Cryptosporidium*-infected HCT-8 cell but can result in adjacent uninfected HCT-8 cell PKCα activation, suggesting a cell-to-cell signaling event during invasion.

To confirm C. parvum activation of intestinal epithelial cell PKCα, we examined this interaction using quantitative immunoblotting. We performed cellular fractionation of HCT-8 cells after treatment with 2.5 nM Gö6976, 200 nM PMA, or C. parvum at an MOI of 4:1. We generated cytoplasmic and membranous fractions and probed all fractions for glyceraldehyde 3-phosphate dehydrogenase (GAPDH) and PKCα. GAPDH localizes to the host cell cytoplasm, is constitutively expressed and stable in HCT-8 cells, and was used to denote the cytoplasmic fraction. We confirmed that pharmacological inhibition with Gö6976 decreased membrane-associated PKCα, while treatment with PMA resulted in increased membrane-associated PKCα (Fig. 1B). After infection with C. parvum, we observed increased membrane-associated PKCα (Fig. 1B). The percentage of PKCα in the membranous fraction after infection with C. parvum is significantly elevated (39.29% ± 1.235% [*P* < 0.0001]) relative to the vehicle controls (Fig. 1C). Next, we asked if the activation of PKCα during C. parvum invasion correlated with the rate of HCT-8 cell infection. To address this, we tested if heat-killed C. parvum sporozoites were capable of activating PKCα (Fig. 1D). Heat-killed C. parvum sporozoites did not induce a significant difference in membrane-associated PKCα relative to the vehicle controls (Fig. 1E). This phenotype remained true when increasing the MOI for heat-killed C. parvum to 20:1 (Fig. 1E). Furthermore, we increased the MOI of live C. parvum to 20:1, compared PKCα in the membranous fraction (Fig. 1D), and found that elevating the MOI resulted in a significant increase in membrane-associated PKCα (19.20% ± 1.902% [*P* = 0.0005]), supporting the activation of PKCα by C. parvum sporozoite invasion (Fig. 1E). Moreover, when comparing membrane-associated PKCα for live C. parvum at an MOI of 4:1 to that at an MOI of 20:1, we observed an additional significant increase (4.522% ± 0.7185% [*P* = 0.0033]) (Fig. 1E). Altogether, these findings show that host intestinal epithelial cell PKCα is activated during C. parvum invasion *in vitro* in two distinct assays.

### A stoplight assay for differentiation of sporozoite adherence from invasion.

To distinguish the earliest steps of C. parvum infection, adherence and invasion, we developed a dual-color “stoplight” immunofluorescence assay (IFA). Antibody labeling of C. parvum-infected HCT-8 cells has been established previously to measure C. parvum development and propagation (37). However, we aimed to characterize sporozoite invasion, which is complete in 2 h *in vitro* after exposure to C. parvum sporozoites (18, 38, 39). To differentiate sporozoite adherence and invasion, we used two identical C. parvum-specific antibodies, conjugated to different fluorophores, coupled with staining before and after cell permeabilization (see Fig. S1A in the supplemental material). This assay was tested using C. parvum-infected HCT-8 cells to determine the assay selectivity for C. parvum sporozoite adherence and invasion. We report two distinct populations of labeled C. parvum-infected HCT-8 cells representative of sporozoite adherence and invasion (Fig. S1B). Adherent C. parvum sporozoites are dually labeled with fluorescein isothiocyanate (FITC)-conjugated Sporo-Glo (a monoclonal antibody that binds to *Cryptosporidium*) and tetramethylrhodamine isothiocyanate (TRITC)-conjugated Sporo-Glo, while invaded sporozoites are singly labeled with FITC-conjugated Sporo-Glo only. We performed single-antibody stains of C. parvum-infected HCT-8 cells separately and observed no spectral overlap between the FITC and TRITC fluorophores (Fig. S2A). Additionally, we stained C. parvum-infected HCT-8 cells with both antibodies simultaneously and observed 100% dually labeled sporozoites, as expected (Fig. S2A). To further evaluate the specificity of our assay to discriminate sporozoite adherence from invasion, we challenged fixed HCT-8 cells with C. parvum sporozoites, which should support only parasite adherence. When fixed HCT-8 cells were exposed to C. parvum, we observed 100% dually labeled C. parvum sporozoites, characteristic of exclusively adherent sporozoites (Fig. S2B). Together, these results validate that our stoplight assay can distinguish C. parvum adherence from invasion.

### Pharmacological inhibition of PKCα decreases Cryptosporidium parvum infection.

**FIG 2:**
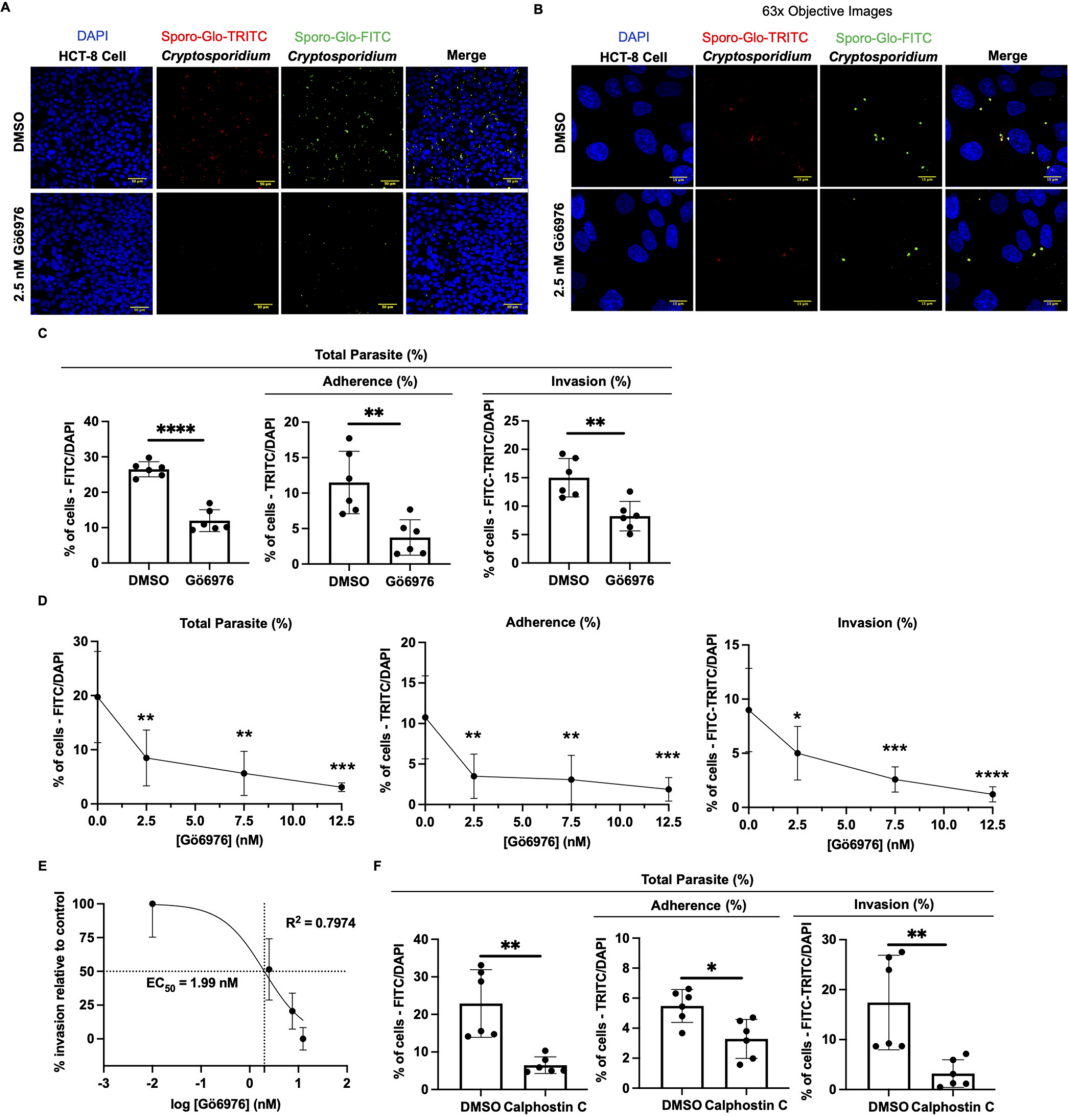
Inhibition of PKCα decreases Cryptosporidium parvum adherence and invasion. (A) Confocal microscopy (20× objective) images of DAPI (HCT-8 cell nucleus), TRITC-conjugated Sporo-Glo (adherent C. parvum), FITC-conjugated Sporo-Glo (total C. parvum), and merged channels. HCT-8 cells were treated with either DMSO (vehicle control) or 2.5 nM Gö6976 for 1 h. (B) High-magnification images (63× objective) denoting differences in C. parvum total parasite number, adherence, and invasion. (C) Bar graph quantification from confocal microscopy images of C. parvum total parasite number and adherence to and invasion of HCT-8 cells after treatment with DMSO or 2.5 nM Gö6976. Data from one of two independent experiments are shown. (D) Concentration curve of treatment with increasing doses of Gö6976 and effect on C. parvum total parasite number, adherence, and invasion relative to the DMSO-treated control. Data from one of two independent experiments are shown. (E) EC_50_ calculation of Gö6976 treatment and C. parvum invasion. Gö6976 concentrations are log transformed, and percent invasion is relative to the DMSO-treated control. (F) Bar graph quantification of C. parvum total parasite number and adherence to and invasion of HCT-8 cells after treatment with DMSO or 50 nM calphostin C for 1 h. Data from one of two independent experiments are shown. For panels C and F, asterisks denote the results of one-tailed unpaired Student’s *t* test (*, *P* < 0.05; **, *P* < 0.01; ***, *P* < 0.001; ****, *P* < 0.00001). For panel D, asterisks denote the results of one-way ANOVA and Tukey’s *post hoc* test (*, *P* < 0.05; **, *P* < 0.01; ***, *P* < 0.001; ****, *P* < 0.00001).

We chose Gö6976 as the primary antagonist for our *in vitro* inhibitor studies due to its previously defined anticryptosporidial activity at PKCα-selective concentrations (29) and because it prevented PKCα activation in uninfected HCT-8 cells (Fig. 1A and B). HCT-8 cells were assessed for Gö6976-induced cytotoxicity using two methods, propidium iodide (PI) staining and HCT-8 cell nucleus counts relative to dimethyl sulfoxide (DMSO)-treated controls (Fig. S3). Using the stoplight assay, we examined the effect of Gö6976 treatment of HCT-8 cells on C. parvum adherence and invasion. HCT-8 cells were treated with 2.5 nM Gö6976 for 1 h prior to infection with C. parvum sporozoites at an MOI of 4:1. At 2 h postinfection, we observed a significant reduction in adherent (FITC-positive [FITC^+^]/TRITC^+^) sporozoites for Gö6976-treated cells relative to the DMSO-treated controls (Fig. 2A). Treatment with Gö6976 reduced the total parasite number by 54.7% (26.5% to 12.0% [*P* < 0.0001]) relative to the DMSO-treated controls, showing that Gö6976 has anticryptosporidial activity early in infection (Fig. 2C). The decrease in the total parasite number corresponded to decreases in sporozoite adherence by 67.5% (11.5% to 4.40% [*P* = 0.0038]) and invasion by 45.1% (15.0% to 8.24% [*P* = 0.003]) (Fig. 2C). At higher concentrations of Gö6976, 7.5 nM and 12.5 nM, we observed reductions in C. parvum total parasite numbers by 71.4% (19.7% to 5.65% [*P* = 0.001]) and 84.4% (19.7% to 3.08% [*P* = 0.0002]), respectively (Fig. 2D). This decrease in total parasite numbers corresponded to decreases in sporozoite adherence by 71.5% (10.8% to 3.07% [*P* = 0.0038]) and 82.6% (10.8% to 1.87% [*P* = 0.0009]) and invasion by 71.3% (8.99% to 2.58% [*P* = 0.0008]) and 86.5% (8.99% to 1.21% [*P* < 0.0001]), respectively (Fig. 2D). We calculated the EC_50_ for Gö6976 inhibition of C. parvum invasion to be 1.99 nM (Fig. 2E), within a close range of the reported IC_50_ of Gö6976 for PKCα (IC_50_ = 2.3 nM) (31). We also evaluated the impact of Gö6976 treatment of C. parvum sporozoites independently and found no significant difference in the total parasite number or adherence to or invasion of HCT-8 cells relative to the DMSO-treated sporozoites (Fig. S4). This suggests that Gö6976 acts primarily via host PKCα inhibition as opposed to other kinases (e.g., PKCβ1 or TrkA) that are targeted by Gö6976 at higher concentrations or a parasite effector.

To further test the importance of PKCα, we used an alternative inhibitor of PKC, calphostin C. Calphostin C induces the inhibition of PKCα through an independent mechanism from Gö6976, and importantly, this inhibition is irreversible, permitting the removal of the compound prior to C. parvum exposure. Calphostin C (50 nM) treatment of HCT-8 cells resulted in a reduction in the C. parvum total parasite number by 71.7% (22.9% to 6.47% [*P* = 0.0015]) relative to the DMSO-treated controls. This total parasite number decrease corresponded to decreases in sporozoite adherence by 40.1% (5.48% to 3.28% [*P* = 0.01]) and invasion by 81.7% (17.4% to 3.19% [*P* = 0.0054]) (Fig. 2F). These data are summarized in Table 1. Altogether, these data support a primary role for host PKCα in mediating both sporozoite adherence and invasion.

**TABLE 1:** Effect of PKCα pharmacological compounds on C. parvum total parasite number and adherence to and invasion of HCT-8 cells^*a*^

Compound	% change (mean % value for control ± SD–mean % value with treatment ± SD [*P* value])		
	Effect on total parasite no.	Effect on adherence	Effect on invasion
Gö6976	↓54.7 (26.5 ± 2.13–12.0 ± 3.09 [<0.0001])	↓67.5 (11.5 ± 4.40–3.74 ± 2.49 [0.0038])	↓45.1 (15.0 ± 3.38–8.24 ± 2.6 [0.003])
Calphostin C	↓71.7 (22.9 ± 9.00–6.47 ± 2.22 [0.0015])	↓40.1 (5.48 ± 1.10–3.28 ± 1.30 [0.01])	↓81.7 (17.4 ± 9.46–3.19 ± 2.76 [0.0054])

PMA	↑149 (26.5 ± 2.13–65.9 ± 5.14 [<0.0001])	↑142 (11.5 ± 4.40–27.8 ± 5.46 [0.0002])	↑154 (15.0 ± 3.38–38.1 ± 8.52 [0.0001])
Bryostatin 1	↑126 (22.9 ± 9.00–51.8 ± 4.63 [<0.0001])	↑225 (5.48 ± 1.10–17.8 ± 4.81 [0.0001])	↑95.4 (17.4 ± 9.46–34.0 ± 4.17 [0.0029])

a For all experiments, unpaired Student’s *t* test was conducted to calculate statistical differences between means of groups of data.

### Pharmacological activation of PKCα increases Cryptosporidium parvum infection.

**FIG 3:**
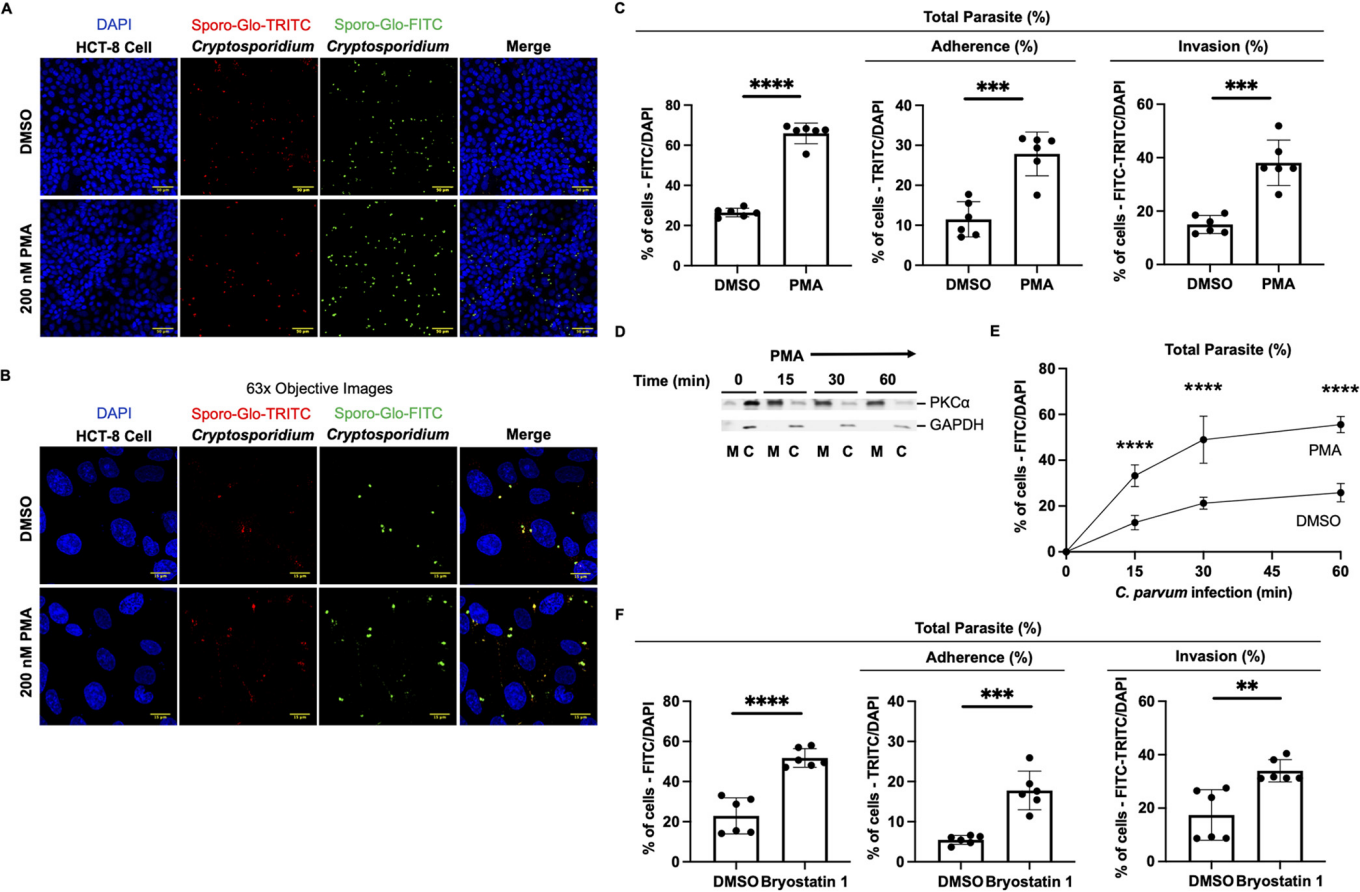
Activation of PKCα increases Cryptosporidium parvum adherence and invasion. (A) Confocal microscopy (20× objective) images of DAPI (HCT-8 cell nucleus), TRITC-conjugated Sporo-Glo (adherent C. parvum), FITC-conjugated Sporo-Glo (total C. parvum), and merged channels. HCT-8 cells were treated with either DMSO or 200 nM PMA for 1 h. (B) High-magnification images (63× objective) denoting differences in C. parvum total parasite number and adherence to and invasion of HCT-8 cells. (C) Bar graph quantification from confocal microscopy images of C. parvum total parasite number and adherence to and invasion of HCT-8 cells after treatment with DMSO or 200 nM PMA. Data from one of two independent experiments are shown. (D) Immunoblots of membrane and cytoplasmic HCT-8 cell fractions treated with 200 nM PMA over time (*n* = 2). (E) Time course of C. parvum infection with or without treatment with 200 nM PMA for 1 h relative to the DMSO-treated control. Data from one of two independent experiments are shown. (F) Bar graph quantification of C. parvum total parasite number and adherence to and invasion of HCT-8 cells after treatment with 100 nM bryostatin 1 or DMSO for 20 min. Data from one of two independent experiments are shown. For panels C and F, asterisks denote the results of one-tailed unpaired Student’s *t* test (*, *P* < 0.05; **, *P* < 0.01; ***, *P* < 0.001; ****, *P* < 0.00001). For panel E, asterisks denote the results of one-way ANOVA and Tukey’s *post hoc* test (*, *P* < 0.05; **, *P* < 0.01; ***, *P* < 0.001; ****, *P* < 0.00001).

We next investigated the impact of the activation of PKCα on C. parvum adherence and invasion. Prior to infection, we activated PKCα by treating HCT-8 cells with PMA (200 nM) for 1 h. HCT-8 cells were assessed for PMA-induced cytotoxicity using PI staining and HCT-8 cell nucleus counts relative to DMSO-treated controls as described above (Fig. S3). Using the stoplight assay, at 2 h postinfection, we observed significant increases in both adherent and invaded (TRITC- and FITC-labeled) C. parvum-infected HCT-8 cells (Fig. 3A and B). PMA-treated HCT-8 cells showed an increase in the C. parvum total parasite number by 149% (26.5% to 65.9% [*P* < 0.0001]) (Fig. 3C). This increased total parasite number corresponded to increases in both sporozoite adherence by 142% (11.5% to 27.8% [*P* = 0.0002]) and invasion by 154% (15.0% to 38.1% [*P* = 0.0001]) relative to the DMSO-treated controls (Fig. 3C). The duration of exposure to PMA has been correlated with the degree of PKCα activation, with extended incubation leading to a paradoxical inhibition caused by ubiquitination and degradation (40, 41). To determine the optimal time required to achieve maximum PKCα activation in HCT-8 cells, we varied the length of exposure to PMA. We observed a rapid translocation of PKCα to the plasma membrane as early as 15 min after treatment with PMA (Fig. 3D). Since PMA-induced activation of PKCα occurred so rapidly, we next examined if priming of PKCα activation accelerates early C. parvum infection. To test this, we challenged HCT-8 cells with C. parvum and quantified the total parasite number at 15 min, 30 min, and 1 h with or without exposure to 200 nM PMA for 1 h. At the earliest recorded time point of 15 min, we observed an increase in the C. parvum total parasite number by 160% (12.8% to 33.3% [*P* < 0.0001]) (Fig. 3E). This increase in the C. parvum total parasite number was significant across all experimental time points relative to the DMSO-treated controls, resulting in increases of 131% (21.2% to 49.0% [*P* < 0.0001]) and 115% (25.9% to 55.6% [*P* < 0.0001]) at 30 min and 1 h, respectively (Fig. 3E). To determine if this increased C. parvum total parasite number was specific to PMA, we introduced an alternative pharmacological agonist of PKCα, bryostatin 1 (42). Bryostatin 1 activates PKC via binding to the C1 domain of PKC, which is distinct from PMA (43). At 2 h postinfection, bryostatin 1 treatment of HCT-8 cells for 20 min resulted in an increase in the C. parvum total parasite number by 126% (22.9% to 51.8% [*P* < 0.0001]) relative to the DMSO-treated controls (Fig. 3F). This increase in the total parasite number corresponded to increases in sporozoite adherence by 225% (5.48% to 17.8% [*P* = 0.0001]) and invasion by 95.4% (17.4% to 34.0% [*P* = 0.0029]). These findings indicate that PKCα activation enhances C. parvum adherence and invasion.

## DISCUSSION

Two major findings emerged from this study. First, C. parvum activated PKCα in human intestinal epithelial cells. Second, host PKCα mediated the initial adherence of parasites as well as internalization resulting in invasion, which we discovered using our stoplight imaging assay. PKCα activity was directly related to parasite infection as pharmacological antagonists blocked C. parvum adherence and invasion, while pharmacological agonists promoted C. parvum adherence and invasion (Table 1). Our studies therefore provide insight into how polymorphisms in human *PRKCA* may increase susceptibility to cryptosporidiosis in children (29), suggesting that increased PKCα activity may increase permissibility to infection at the intestinal epithelium. We now have shown a novel role for host PKCα in *Cryptosporidium* adherence to and invasion of human intestinal epithelial cells. The facts that *Cryptosporidium* targets intestinal epithelial cell PKCα during early infection and PKCα activity is critical for a pathogenic host-mediated cellular process make it an attractive target for host-directed therapy.

Pharmacological inhibition of intestinal epithelial cell PKCα reduced, while activation of PKCα enhanced, C. parvum adherence and invasion. These findings implicate host PKCα activity in promoting both parasite adherence and invasion. Importantly, we observed that treatment with the competitive inhibitor Gö6976 and the irreversible antagonist calphostin C resulted in comparable reductions in adherence and invasion. Mechanistically, these compounds alter PKCα through dissimilar actions. Gö6976 inhibits PKCα through competition with ATP (31), while calphostin C inhibits PKCα through C1 domain binding (44, 45). The use of pharmacological antagonists to target PKCα with differing mechanisms of action further supports a role for PKCα-mediated parasite entry. Moreover, C. parvum sporozoites showed no significant difference in infection of intestinal epithelial cells upon treatment with Gö6976. This finding was expected as no known homologs of PKCα have been reported in any *Cryptosporidium* spp., adding additional support for a host-inhibitory effect. We additionally found that treatment with a PKCα agonist alters the kinetics of infection *in vitro*. Altogether, these findings confirm a role for PKCα in parasite adherence and invasion and suggest the activation of PKCα as a preliminary event to C. parvum infection.

PKCα alters the morphology of the host cell F-actin cytoskeleton, thereby regulating processes that are affected by the reorganization of these microfilaments (46, 47). PKCα is active at the cell membrane, the same site as parasite adherence and invasion. Hence, it is biologically plausible for PKCα to have activity at the host-parasite interface. Two kinases important for regulating actin polymerization have been implicated in the required rearrangement of F-actin during *Cryptosporidium* infection: proto-oncogene tyrosine-protein kinase Src (c-Src) and phosphatidylinositol 3-kinase (PI3K) (20, 48–50). Inhibition of c-Src via a dominant negative mutant blocked C. parvum-mediated rearrangement of F-actin and, subsequently, C. parvum invasion of human biliary epithelial cells (48). Similarly, genetic and pharmacological inhibition of PI3K decreased C. parvum-induced actin rearrangements and blocked invasion of human biliary epithelial cells (50). PKCα has been reported upstream to regulate c-Src (51, 52) and PI3K (53, 54). Although these studies did not discern parasite adherence from invasion, we speculate that sporozoite adherence results in the activation and recruitment of host PKCα to the cell membrane. This results in a PKCα phosphorylation cascade leading to the F-actin remodeling required for invasion (see Fig. S5 in the supplemental material). This research furthers our current understanding of the anticryptosporidial activity of PKCα antagonists by pinpointing activity to specific life cycle stages. Furthermore, we have established C. parvum-induced activation of PKCα as a critical event during invasion. Thus, PKCα is a key host protein target meriting further investigation as its activity will yield additional insight into the molecular mechanisms of parasite invasion.

Host-directed therapy is an emerging approach in the field of anti-infectives. Recent insights into pathogen-host interactions are leading to the identification of a wide array of host targets involved in pathogenesis. We show here that PKCα activity mediates *Cryptosporidium* adherence to and invasion of intestinal epithelial cells *in vitro*. Evidence from our *in vitro* analyses suggests that PKCα is a potential host target for therapy in human infection with *Cryptosporidium*, although further experimentation is necessary, in particular as our analysis examined only the addition of inhibitors prior to infection with sporozoites. Remarkably, there remains no PKCα-specific FDA-approved drug despite isozyme-specific contrasts in activity being linked to a number of human disease states (55–58). However, the PKC agonist bryostatin 1 is currently in phase 2 clinical trials for the treatment of moderately severe to severe Alzheimer’s disease (59). Consequently, the pharmacological antagonists used in this study may serve as suitable options for future anticryptosporidial studies.

Using our fluorescence-based approach to examine PKCα activity, we observed the activation of PKCα in not only *Cryptosporidium*-infected cells but also adjacent uninfected intestinal epithelial cells. Our pharmacological data suggest that the host cell baseline PKCα activation state may determine hospitality to *Cryptosporidium* infection. Therefore, the activation of PKCα in adjacent uninfected intestinal epithelial cells may have biological relevance. We speculate that PKCα activation in adjacent uninfected cells is the result of a cell contact signaling event. A host pathway for PKC activation has been found for other protozoan parasites during invasion (60). Entamoeba histolytica and *Cryptosporidium* spp. recognize the same surface sugar for adherence to human cells. We hypothesize that *Cryptosporidium* sporozoite adherence to an intestinal epithelial cell induces a rapid influx of calcium ions (Ca^2+^) and subsequently in adjacent uninfected cells through cellular gap junctions. The intracellular Ca^2+^ influx activates host PKCα in *Cryptosporidium*-infected cells and adjacent uninfected cells. Delineating intracellular Ca^2+^ signaling and PKCα activation during epithelial cell *Cryptosporidium* infection is an important next step.

This study directly builds on the identification of *PRKCA* in a forward genetic screen by defining a role experimentally, at an enzymatic level, for susceptibility in human intestinal epithelial cells *in vitro*. Our pharmacological analyses suggest that the previously identified polymorphisms in *PRKCA* would increase PKCα activity, via increased mRNA expression or protein stability. Furthermore, these data suggest that the polymorphisms associated with increased susceptibility act through exerting changes in the intestinal epithelium. Expression quantitative trait locus (eQTL) analyses have linked the associated SNPs with decreased *PRKCA* mRNA expression in the esophagus and colon (61). However, the impact on small intestinal expression, which is the major site of *Cryptosporidium* infection, has not been determined. Our findings suggest that SNPs associated with increased susceptibility to *Cryptosporidium* might act via increased PKCα activity during *Cryptosporidium* infection, either by increasing *PRKCA* mRNA expression or via compensatory gene regulation of PKCα regulators. Alternatively, the activation of human intestinal cell PKCα may be *Cryptosporidium* specific, resulting in altered expression only upon exposure to parasites.

A limitation of the *in vitro* system is that HCT-8 cells are cancer cells and may have aberrant PKCα activity (62). This concern is somewhat mitigated by the observation that PKCα was activated and inhibited as expected in HCT-8 cells. An additional limitation of our system is the inability to investigate the immune response to infection. Th17 cells have been implicated in the response to infection, with increased levels of Th17-related cytokines being reported in the gut (63), and PKCα is a positive regulator of Th17 cell function (64). Moreover, gut infection has been linked to immune-mediated alterations leading to functional changes in the intestinal epithelium (65). Here, modifications of intestinal epithelial cell actin-binding proteins, ezrin and villin, played a role in Giardia pathophysiology and are CD4^+^ and CD8^+^ T cell dependent (65). To address a secondary role for PKCα in the immune response to *Cryptosporidium* infection, further examination *in vivo* is needed. A major strength of this study is the identification of intestinal epithelial cell PKCα activation by C. parvum, which implicates yet another host kinase in infection. Second, we used a well-characterized intestinal cell model that allowed the examination of PKCα activity at precise stages of parasite infection. The use of this intestinal cell system also allowed the investigation of PKCα devoid of the influence of immune cell regulation. Finally, the introduction of the stoplight assay for the differentiation of parasite adherence from invasion and downstream quantification is an asset for future studies in the field.

In conclusion, using an *in vitro* system of intestinal epithelial cell cryptosporidiosis, we expand on the role of PKCα and susceptibility to infection. We elucidated the parasite stages in which PKCα is involved by demonstrating that changes in PKCα activity impact downstream C. parvum adherence and invasion. We show in this study that C. parvum sporozoites activate intestinal epithelial cell PKCα and prove that PKCα activation is critical for C. parvum adherence and invasion. These results lead us to conclude that the activation of PKCα by C. parvum is deliberate and an important component of infection of intestinal epithelial cells. Thus, the mechanisms by which PKCα promotes C. parvum invasion and its role *in vivo* warrant further exploration.

## MATERIALS AND METHODS

### HCT-8 intestinal epithelial cell culture.

Human ileocecal adenocarcinoma (HCT-8; ATCC CCL 244) cells were purchased from the American Type Culture Collection. Cells were maintained in T-75 (75-cm^2^) tissue culture flasks as adherent monolayers in growth medium consisting of RPMI 1640 medium (catalog number 11875093) supplemented with l-glutamine and 10% heat-inactivated fetal bovine serum (FBS). Cells were stored at 37°C in a 5% CO_2_ humidified incubator until confluence. One day prior to infection, cells were washed once with 1× phosphate-buffered saline (PBS), collected at 37°C with 0.25% trypsin-EDTA, and resuspended in growth medium. Cells were then plated (500 μL/well) into 24-well assay plates at a density of 1.14 × 10^5^ cells/well. Cells were allowed to grow for 24 h at 37°C with 5% CO_2_ in a humidified incubator.

### Cryptosporidium parvum oocysts.

Oocysts of Cryptosporidium parvum (Iowa strain) were purchased from Waterborne, Inc., and stored at 4°C for ≤3 months in PBS with antibiotics (penicillin, streptomycin, amphotericin B, gentamicin, and 0.01% Tween 20).

### Pharmacological compounds.

Four compounds were used to modulate PKCα activity prior to Cryptosporidium parvum infection *in vitro*. All compounds were commercially sourced from reputable vendors and supplied as high-purity (≥95%) solids. Gö6976 and PMA were purchased from Abcam. Calphostin C was purchased from Calbiochem. Bryostatin 1 was purchased from Sigma-Aldrich. Compounds were prediluted in dimethyl sulfoxide (DMSO) at 1 mg/mL. All experiments using pharmacological compounds were performed in biological triplicate.

### C. parvum infection of HCT-8 cells.

Oocysts were excysted 24 h after seeding of HCT-8 cells, as described previously (66). Briefly, the oocysts were centrifuged at 16,000 × *g* for 3 min in a microcentrifuge at 4°C. Oocysts were then incubated in a 1:4 bleach solution on ice for 5 min and then centrifuged at 16,000 × *g* for 3 min at 4°C. The supernatant was gently aspirated, and the oocysts were resuspended in 1 mL of 1× PBS and then centrifuged for a total of three washes at 4°C. After the final wash, the supernatant was gently removed, and the oocysts were resuspended in 0.5 mL of 0.75% sodium taurocholate and incubated at 15°C for 10 min. After incubation in bile salts, the oocysts were stored in a 37°C heat block and incubated for 1 h. For heat-killed C. parvum, sporozoites from excysted oocysts were then incubated at 75°C for 60 s (67). After incubation, the oocysts were centrifuged at 3,000 × *g* for 1 min and resuspended in 0.5 mL of assay medium consisting of RPMI 1640 medium without phenol red (catalog number 11835030) supplemented with 2% heat-inactivated FBS. Oocysts were then enumerated using a disposable hemocytometer to determine the percentage of sporozoite excystation. Growth medium was gently removed from the 24-well assay plate of seeded HCT-8 cells and replaced with 0.5 mL of assay medium. Sporozoites from excysted oocysts were added to each well (5.7 × 10^5^ sporozoites/well) of the 24-well assay plate. Plates were then spun at 150 × *g* for 3 min in a benchtop centrifuge at room temperature. Infected cells were incubated at 37°C with 5% CO_2_ in a humidified incubator for 2 h.

### Stoplight immunofluorescence assay for C. parvum adherence and invasion.

After infection, assay medium containing infectious parasites was aspirated, and cells were washed with prewarmed assay medium to remove unbound parasites. Cells were then fixed with 4% paraformaldehyde for 15 min at room temperature, followed by 3 washes with 1× PBS for 3 min at room temperature. To prevent nonspecific binding, the cells were blocked with Dilution/Blocking (DB) buffer (Waterborne, Inc.) for 30 min at room temperature. Cells were stained with TRITC-conjugated Sporo-Glo (Waterborne, Inc.) diluted 1:20 in DB buffer for 1 h at room temperature protected from light. Cells were washed three times with 1× PBS supplemented with 0.01% Tween 20 (1× PBST) for 3 min. After washing, each well was permeabilized with 0.1% Triton X-100 diluted in 1× PBS for 15 min at room temperature and then washed 3 times with 1× PBST. Cells were then stained with FITC-conjugated Sporo-Glo (Waterborne, Inc.) diluted 1:20 in DB buffer for 1 h at room temperature protected from light. Cells were washed three times with 1× PBST for 3 min. To visualize HCT-8 cell nuclei, cells were stained with 4′,6-diamidino-2-phenylindole (DAPI) diluted 1:1,000 in 1× PBS for 10 min at room temperature protected from light. Finally, cells were washed 3 times with 1× PBST. The cells were imaged with a Zeiss LSM 700 confocal microscope (Carl Zeiss, Inc.) using a 20× objective. Three channels were used: 405 nm for DAPI-stained nuclei, 488 nm for FITC-labeled *Cryptosporidium* parasites, and 543 nm for TRITC-labeled *Cryptosporidium* parasites.

For each assay (pharmacological inhibition/activation, dose response, and time course), 2 fields were captured for each well in biological triplicate. ZEN 2.1 Black Edition software was used to obtain z-stacks through the entire height of the cells with confocal z-slices of 1.0 μm (z-stack = 26 μm). HCT-8 cytotoxicity (number of nuclei relative to DMSO-treated controls) and contrasts in *Cryptosporidium* sporozoite adherence and invasion (cell counts relative to DMSO-treated controls) were determined. Images were processed using Fiji Is Just ImageJ (FIJI) software (version 2.0.0-rc-41/1.50d). HCT-8 cell nuclei were counted using the analyze particles command selecting for units 50 to 1,000 μm^2^ in size. TRITC-labeled *Cryptosporidium* parasites and FITC-labeled *Cryptosporidium* parasites were counted using the analyze particles command selecting for units 2 to 20 μm^2^ in size.

### Immunofluorescence assay for PKCα localization.

Assay medium containing infectious parasites was aspirated, and cells were washed with prewarmed assay medium to remove unbound parasites. Cells were then fixed with 4% paraformaldehyde for 15 min at room temperature. Each well was then washed three times with 1× PBS for 3 min at room temperature. After washing, each well was permeabilized with 0.1% Triton X-100 diluted in 1× PBS for 15 min at room temperature and then washed three times with 1× PBS. To prevent nonspecific binding, the cells were blocked with DB buffer for 30 min at room temperature. C. parvum parasites were then stained with FITC-conjugated Sporo-Glo diluted 1:20 in DB buffer for 1 h at room temperature protected from light. Cells were washed three times with 1× PBST for 3 min. HCT-8 cell PKCα was stained with an anti-PKCα antibody (catalog number ab32376; Abcam) used at a dilution of 1:200 in 1× PBS supplemented with 1% bovine serum albumin (BSA) for 1 h at room temperature. A secondary goat anti-rabbit Cy3 antibody (catalog number ab6939; Abcam) was used at a dilution of 1:1,000 in 1× PBS supplemented with 1% BSA for 1 h at room temperature protected from light. Cells were washed three times with 1×PBST for 3 min. HCT-8 cell nuclei were stained with DAPI diluted 1:1,000 in 1×PBS for 10 min at room temperature protected from light. Finally, cells were washed three times with 1× PBST and covered. The 24-well plates were imaged on a Zeiss LSM 700 confocal microscope (Carl Zeiss, Inc.) with a 63× objective with immersion oil. Three channels were used: 405 nm for DAPI-stained nuclei, 488 nm for FITC-labeled *Cryptosporidium* parasites, and 543 nm for Cy3-labeled PKCα. For each well, 2 fields were captured. ZEN 2.1 Black Edition software was used to obtain z-stacks through the entire height of the cells with confocal z-slices of 1.0 μm (z-stack = 16 μm). HCT-8 cytotoxicity (number of nuclei relative to DMSO-treated controls) was determined. Images were processed using FIJI software.

### Cell fractionation and immunoblot analysis.

After the completion of the infection, cells were washed with prewarmed assay medium to remove unbound parasites. Each well containing *Cryptosporidium*-infected cells was treated with 0.25% trypsin-EDTA for 10 min at 37°C and collected into Eppendorf tubes. A small aliquot of cells was removed for enumeration, and the remaining cells were pelleted at 300 × *g* for 10 min. After centrifugation, cell fractionation was performed by using a commercially available cell fractionation kit (catalog number ab109719; Abcam) according to the manufacturer’s instructions. Briefly, cells were resuspended in 0.1 mL of 1× buffer A (Abcam) supplemented with a 1× Halt protease and phosphatase inhibitor cocktail (Thermo Fisher). Cells were permeabilized with detergent I (Abcam) and then pelleted at 5,000 × *g* for 2 min and again at 10,000 × *g* for 2 min at 4°C. The resultant supernatant was collected as the cytoplasmic fraction. The cytoplasm-depleted pellet was resuspended in 0.1 mL of 1× buffer A. The protein concentration in each fraction was quantified using a Qubit fluorometer (Thermo Fisher). Samples were prepared 1:1 with 2× Laemmli sample buffer (Bio-Rad) and boiled for 5 min at 100°C. Samples were run on an Any kD Mini-Protean TGX polyacrylamide gel (Bio-Rad), transferred to a nitrocellulose membrane, and blocked with Intercept (PBS) blocking buffer (Li-Cor Biosciences). Anti-PKCα antibody was used at a dilution of 1:1,000. Secondary donkey anti-rabbit IR dye 680 (Li-Cor Biosciences) was used at a dilution of 1:10,000. Anti-GAPDH antibody (catalog number 97166; Cell Signaling Technology) was used at a dilution of 1:1,000. Secondary goat anti-mouse IR dye 800 (Li-Cor) was used at a dilution of 1:10,000. Blots were imaged with a Li-Cor Biosciences Odyssey imaging system. Images were analyzed using the analysis feature in ImageStudioLite software (version 5.2.5; Li-Cor Biosciences). The band intensity for PKCα under each experimental condition was measured using the analysis feature in ImageStudioLite software (version 5.2.5; Li-Cor Biosciences). PKCα activation (percentage of PKCα in the membrane fraction relative to the cytoplasmic fraction) was calculated under each condition relative to the DMSO-treated vehicle controls.

### HCT-8 cell cytotoxicity.

HCT-8 cells were assessed for the cytotoxicity of pharmacological compounds using two methods, PI staining and HCT-8 cell nucleus counts relative to DMSO-treated controls. In each case, cells were seeded (500 μL/well) into 24-well assay plates at a density of 1.14 × 10^5^ cells/well. Cells were exposed to each experimentally matched concentration of pharmacological compounds resuspended in assay medium. Cells were incubated at 37°C with 5% CO_2_ in a humidified incubator for 2 h. After incubation, cells were exposed to a PI-RNase A solution (Phoenix Flow Systems, Inc.). After a 30-min incubation at room temperature, the live cells were imaged on a Zeiss LSM 700 confocal microscope with a 20× objective. The number of PI-labeled cells for exposure to each compound was calculated relative to a maximum cytotoxicity control. For further confirmation, the total number of nuclei per field for compound-exposed cells relative to DMSO-treated controls was measured.

### Data and statistical analyses.

Prism 9 (GraphPad Software, La Jolla, CA) was used for the generation of graphs and statistical analysis. Image quantification was evaluated for significance using unpaired Student’s *t* test or one-way analysis of variance (ANOVA) and Tukey’s *post hoc* test to determine statistical differences between means of groups of data from all tissue culture experiments. All data show the results from 1 of 2 independent experiments in biological triplicate.
