# Supplementary material for: Protein Kinase C-α Is a Gatekeeper of Cryptosporidium Sporozoite Adherence and Invasion
Source: Infect Immun. 2022 Mar 17;90(3):e00679-21. doi: 10.1128/iai.00679-21 (PMC8929341; doi:10.1128/iai.00679-21)
Supplement: Supplemental file 1 — Supplemental material. Download iai.00679-21-s0001.pdf, PDF file, 702 KB [file iai.00679-21-s0001.pdf]

SUPPLEMENTARY FIGURE 1

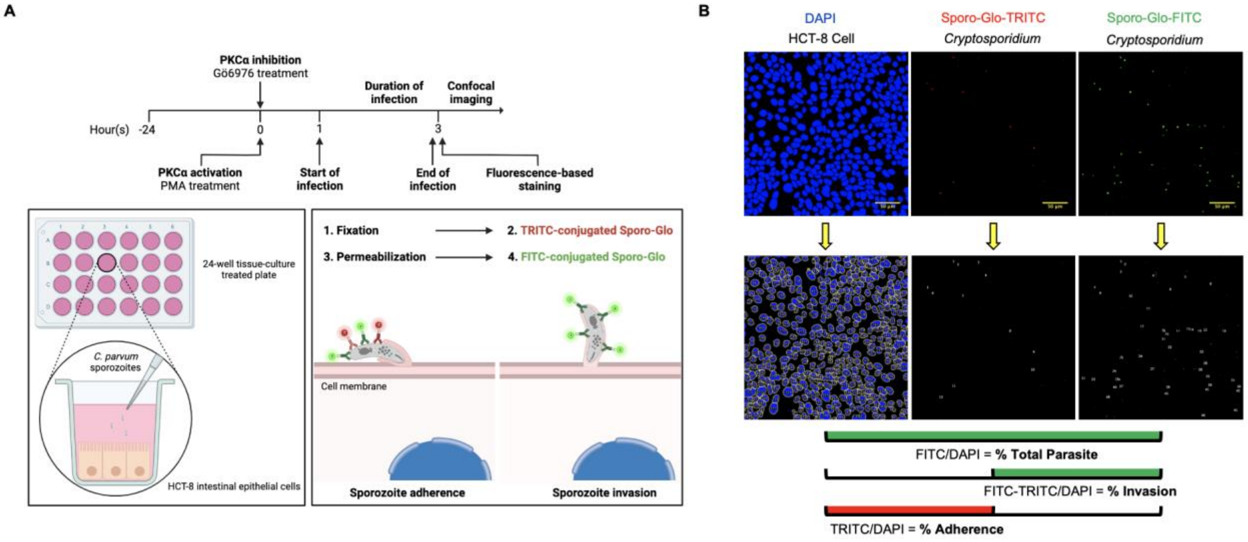

SUPPLEMENTARY FIGURE 2

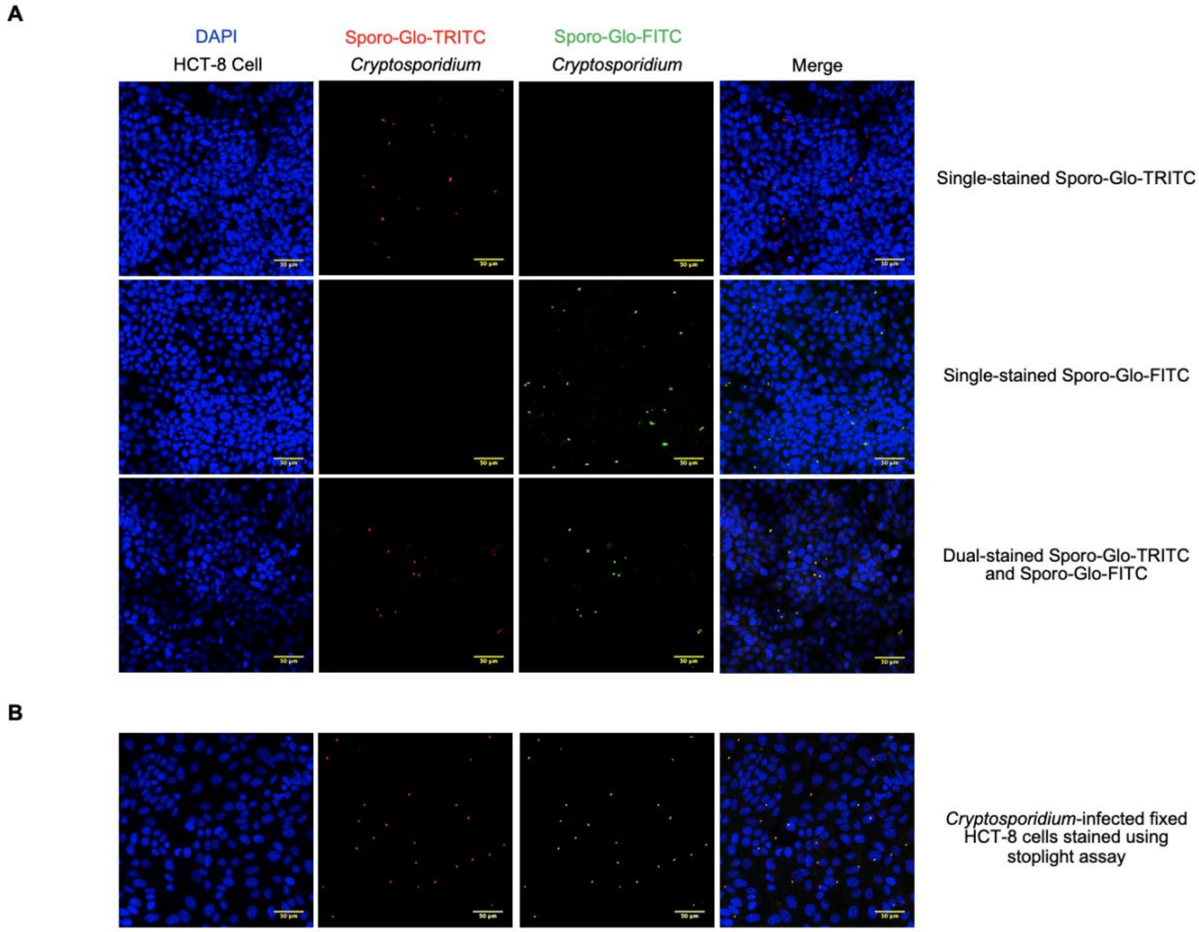

SUPPLEMENTARY FIGURE 3

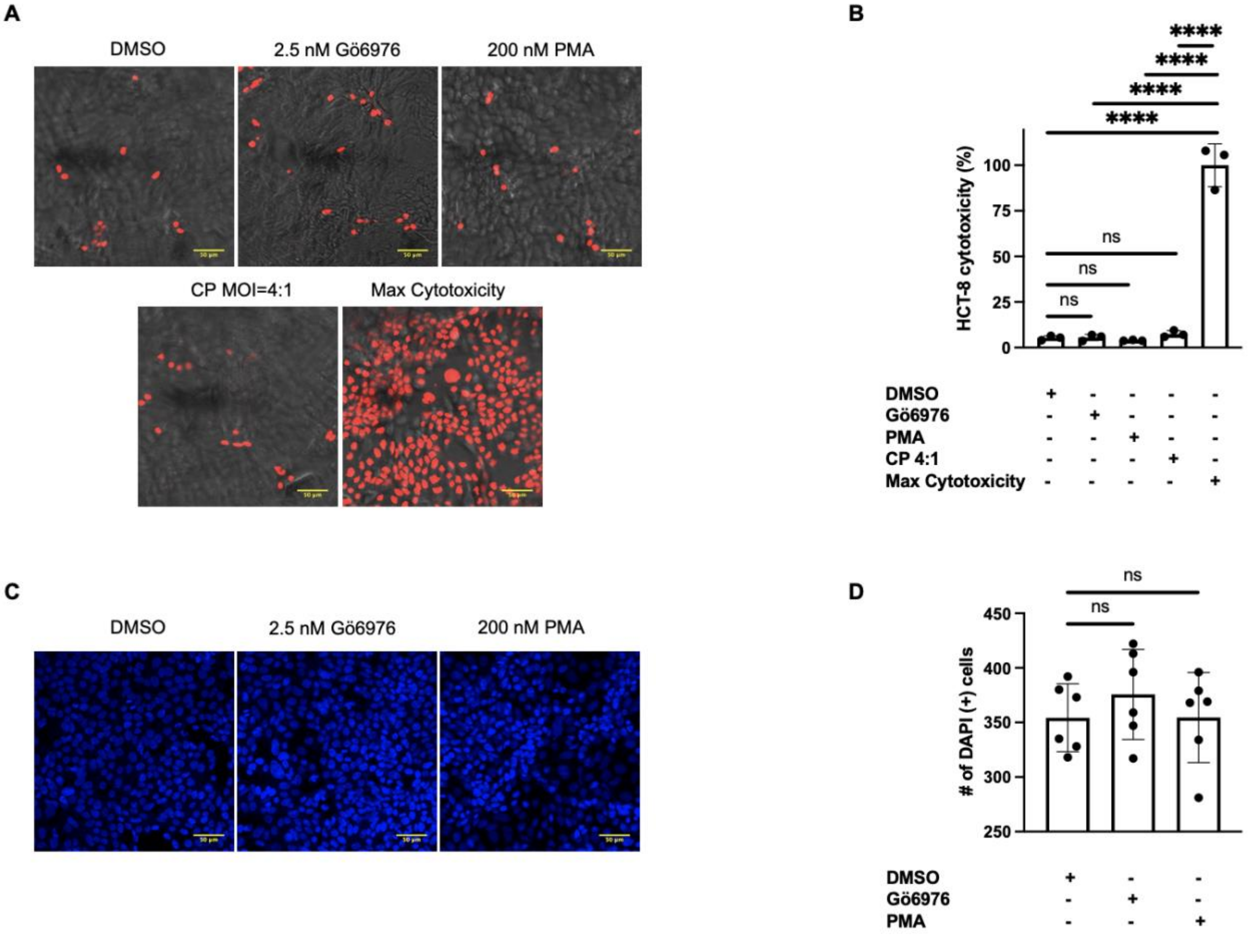

SUPPLEMENTARY FIGURE 4

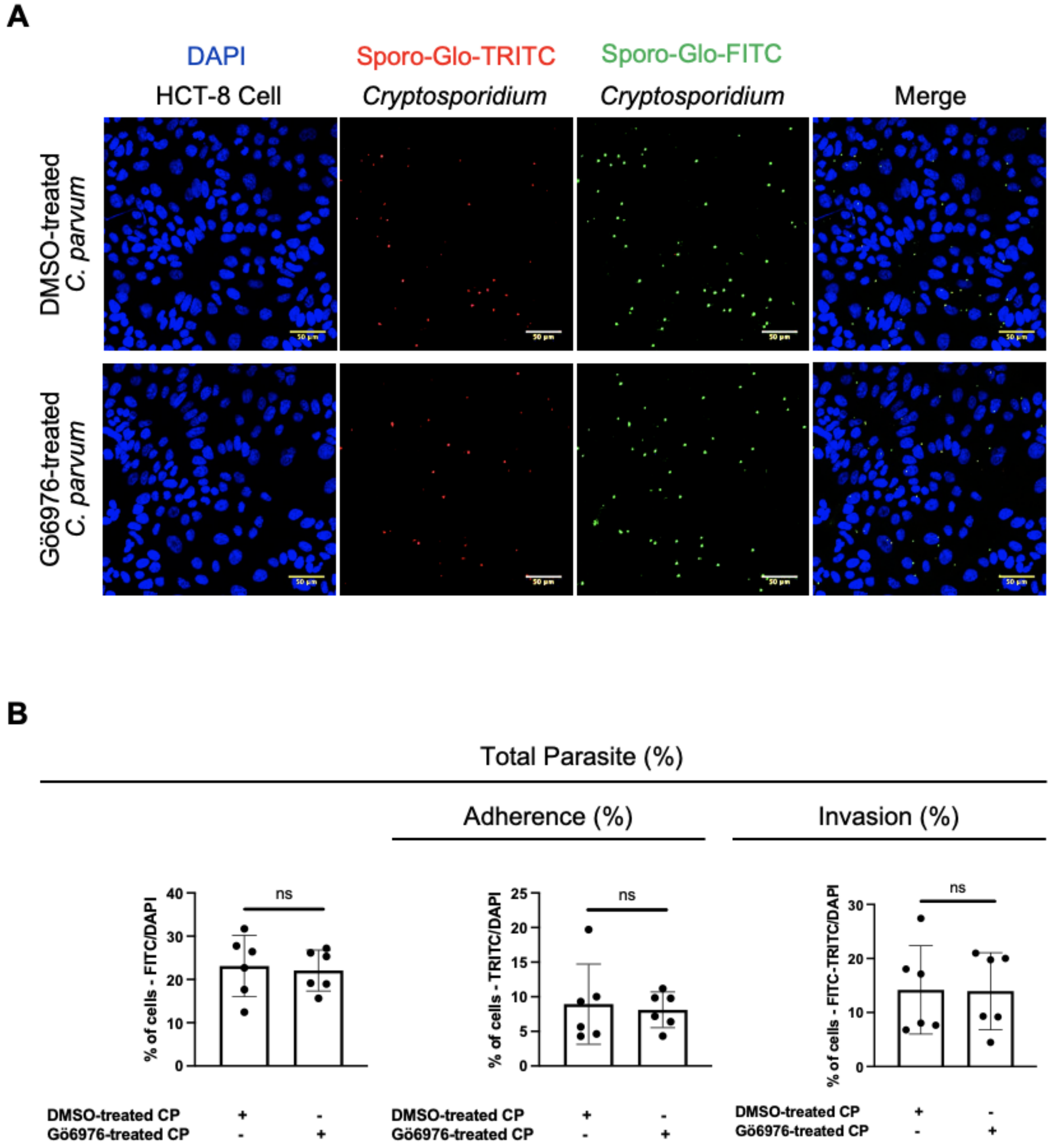

SUPPLEMENTARY FIGURE 5

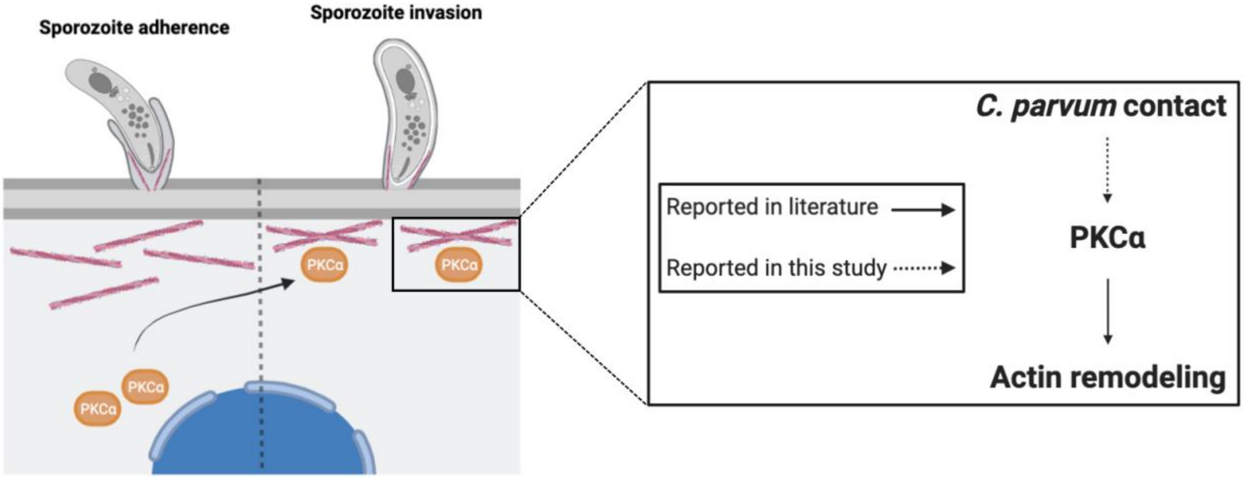

FIGURE AND TABLE LEGENDS

SUPPLEMENTARY FIGURE 1. Imaging "stoplight" assay to differentiate *C. parvum* adherence from invasion *in vitro*. (A) HCT-8 intestinal epithelial cells were seeded into a 24-well tissue culture treated plate. 24 h later, each well containing HCT-8 cells were treated with a PKCα pharmacologic inhibitor or activator to alter PKCα activity. Next, cells were challenged with *C. parvum* at a MOI of 4:1 for 2 h. After infection, *Cryptosporidium*-infected HCT-8 cells were fixed with 4% paraformaldehyde then stained with a TRITC-conjugated Sporo-Glo antibody specific to *Cryptosporidium*. Next, the *Cryptosporidium*-infected HCT-8 cells were permeabilized with 0.1% Triton X-100 and subsequently stained with a FITC-conjugated Sporo-Glo antibody. This method of staining produces two populations of labeled *Cryptosporidium*, dual-labeled (TRITC+/FITC+) extracellular sporozoites and single-labeled (FITC+) intracellular sporozoites. (B) Confocal microscopy (20x objective) images of DAPI (HCT-8 cell nucleus), TRITC-conjugated Sporo-Glo (adherent *C. parvum*), FITC-conjugated Sporo-Glo (total *C. parvum*). Quantification of each image using spot counts are shown below with numerical values indicating a positive event. Calculations for *C. parvum* total parasite number, adherence, and invasion were performed using recorded spot counts for FITC/DAPI, TRITC/DAPI, and FITC-TRITC/DAPI, respectively.

SUPPLEMENTARY FIGURE 2. Evaluating stoplight assay for fluorescent overlap and specificity. (A) HCT-8 intestinal epithelial cells were seeded into a 24-well tissue culture treated plate. 24 h later, each well containing HCT-8 cells were challenged with

C. parvum at a MOI of 4:1 for 2 h. After infection, *Cryptosporidium*-infected HCT-8 cells were fixed with 4% paraformaldehyde then permeabilized with 0.1% Triton X-100. Next, the *Cryptosporidium*-infected HCT-8 cells were stained with either the TRITC-conjugated Sporo-Glo antibody, the FITC-conjugated Sporo-Glo antibody, or both. (B) HCT-8 intestinal epithelial cells were seeded into a 24-well tissue culture treated plate. 24 h later, each well containing HCT-8 cells was fixed with 4% paraformaldehyde then challenged with *C. parvum* at a MOI of 4:1 for 2 h. Each well was then fixed again with 4% paraformaldehyde then stained with the TRITC-conjugated Sporo-Glo antibody. Lastly, the *Cryptosporidium*-infected fixed-HCT-8 cells were permeabilized with 0.1% Triton X-100 then stained with the FITC-conjugated Sporo-Glo antibody.

SUPPLEMENTARY FIGURE 3. Evaluating HCT-8 intestinal epithelial cells for pharmacologic cytotoxicity. (A) Intestinal epithelial cells were treated with DMSO, 2.5 nM Gö6976, 200 nM PMA, or *C. parvum* at a MOI of 4:1. To achieve maximum cytotoxicity, HCT-8 intestinal epithelial cells were fixed with 4% paraformaldehyde. Confocal fluorescence microscopy images of propidium iodide (PI) stained HCT-8 cells and differential interference contrast (DIC) are shown. (B) Bar graph quantification of HCT-8 cell cytotoxicity after exposure to DMSO, pharmacologic compounds, *C. parvum*, or 4% paraformaldehyde. (C) Confocal microscopy (20x objective) images of DAPI (HCT-8 cell nucleus) stained cells after treatment with DMSO, 2.5 nM Gö6976, or 200 nM PMA. (D) Bar graph quantification of HCT-8 cell cytotoxicity after exposure to DMSO or pharmacologic compounds. For PI experiments, asterisks denote results of one-way ANOVA and Tukey's post-hoc test used to analyze percent (%) cytotoxicity

(*P<0.05;**P<0.01;***P<0.001;****P<0.00001). When comparing HCT-8 cell nuclei counts to determine percent (%) cytotoxicity, asterisk denote results of unpaired Student's t test (*P<0.05;**P<0.01;***P<0.001;****P<0.00001).

SUPPLEMENTARY FIGURE 4. Gö6976-treated *C. parvum* sporozoites exhibit no difference in infection of HCT-8 intestinal epithelial cells. (A) Confocal microscopy (20x objective) images of DAPI (HCT-8 cell nucleus), TRITC-conjugated Sporo-Glo (adherent *C. parvum*), FITC-conjugated Sporo-Glo (total *C. parvum*), and merged channels. *C. parvum* sporozoites were either treated with DMSO (vehicle control) or 2.5 nM Gö6976 for 1 h. (B) From confocal microscopy images, bar graph quantification of *C. parvum* total parasite number, adherence, and invasion of HCT-8 cells after treatment with DMSO or 2.5 nM Gö6976. Asterisks denote results of one-tailed unpaired Student's t-test (*P<0.05;**P<0.01;***P<0.001;****P<0.00001).

SUPPLEMENTARY FIGURE 5. Schematic model for PKCα-induced actin remodeling during *Cryptosporidium* invasion. *Cryptosporidium* contact results in activation and recruitment of host intestinal epithelial cell PKCα to the plasma membrane. Activated PKCα can function at the host-parasite interface to remodel host cell F-actin required for *Cryptosporidium* invasion.
